# Swine Influenza Virus A (H3N2) Infection in Human, Kansas, USA, 2009

**DOI:** 10.3201/eid1706.101488

**Published:** 2011-06

**Authors:** Chad M. Cox, Daniel Neises, Rebecca J. Garten, Bill Bryant, Richard A. Hesse, Gary A. Anderson, Ingrid Trevino-Garrison, Bo Shu, Stephen Lindstrom, Alexander I. Klimov, Lyn Finelli

**Affiliations:** Author affiliations: Centers for Disease Control and Prevention, Atlanta, Georgia, USA (C.M. Cox, R.J. Garten, B. Shu, S. Lindstrom, A.I. Klimov, L. Finelli);; Kansas Department of Health and Environment, Topeka, Kansas, USA (D. Neises, I. Trevino-Garrison);; Kansas Animal Health Department, Topeka (B. Bryant);; Kansas State Veterinary Diagnostic Laboratory, Manhattan, Kansas, USA (R.A. Hesse, G.A. Anderson)

**Keywords:** Swine, origin, influenza, virus, human, triple, reassortant, H3N2, letter

**To the Editor:** Triple-reassortant swine influenza viruses (SIVs), which contain genes from human, swine, and avian influenza A viruses, have been enzootic among swine herds in the United States since the late 1990s (*1*). Although uncommon, occasional transmission of triple-reassortant SIVs from swine to humans has occurred (*2**–**4*). Before April 2009, only limited, nonsustained human-to-human transmission of SIVs had been reported (*5**–**7*). Although an animal source for pandemic (H1N1) 2009 virus has yet to be identified, the pandemic strain resulted from the reassortment of 2 different lineages of SIV (*8*).

On July 28, 2009, a 12-year-old Kansas boy sought treatment for fever, cough, and sore throat. Results of an influenza rapid antigen test were positive, and a specimen was sent to the Kansas Department of Health and Environment for further testing. Real-time reverse transcription PCR (rRT-PCR) testing determined the virus contained the surface hemagglutinin (HA) gene of influenza A (H3) and the internal nucleoprotein gene common to all triple-reassortant SIVs (*9*). The specimen was sent to the Centers for Disease Control and Prevention (Atlanta, GA, USA) on August 3 and identified as swine-origin influenza virus A (H3N2) by rRT-PCR and sequence analysis.

The patient reported that during July 23–25, 2009, he touched healthy-appearing swine multiple times while attending a county fair. The boy received a standard treatment course of oseltamivir and recovered completely. None of his 3 household contacts attended the fair, and none reported signs or symptoms of illness in the weeks afterward.

The Kansas Department of Health and Environment and the local health department collaborated with the county extension office to identify and interview swine exhibitors at the county fair, focusing on influenza symptoms among exhibitors and household contacts during the week before and after the fair. Twenty-seven (79%) of 34 exhibitors participated in the survey; none reported signs or symptoms of influenza-like illness, defined as fever (temperature >100°F) accompanied by either cough or sore throat. Two household contacts of separate exhibitors each reported a low-grade fever (<100°F) and sore throat in the week after the fair. Both touched swine while attending the fair. Both visited a physician’s office; neither was tested for influenza; and symptoms of both resolved without treatment. The veterinarian overseeing the swine barn reported no signs of respiratory illness among the swine during the fair. Most swine exhibited were slaughtered at the fair’s conclusion.

On August 7, the Kansas Animal Health Department collected nasal swab specimens and blood samples from 13 swine belonging to 7 exhibitors. All samples were delivered to the Kansas State Veterinary Diagnostic Laboratory for analysis by influenza matrix rRT-PCR and virus isolation on nasal swab samples and hemagglutination inhibition (HI) assays against classical swine influenza virus (H1N1) (A/swine/Iowa/73) and the prototype swine influenza virus (H3N2) (A/swine/Texas/98) on serum samples. In addition, the influenza virus (H3N2) “county fair” isolate, A/Kansas/13/2009 (H3N2), was sent from the Centers for Disease Control and Prevention, amplified, and used to develop a second HI assay that included the original swine serum samples as well as paired convalescent-phase samples from 3 of the swine (Table).

**Table:** Hemagglutination inhibition assay titers for 3 influenza strains from swine exhibited at county fair, by date of blood draw, Kansas, 2009*

Swine ID no.	August 7 titers				August 31 titers	
	Subtype H1N1†	Subtype H3N2‡	“County fair,” subtype H3N2§		Subtype H3N2‡	“County fair,” subtype H3N2§
1	<10	20	320		¶	¶
2	<10	10	160		¶	¶
3	<10	40	640		¶	¶
4	<10	40	640		¶	¶
5	<10	80	640		¶	¶
6	<10	40	640		¶	¶
7	<10	320	640		¶	¶
8	10	10	160		¶	¶
9	<10	80	640		¶	¶
10	<10	40	320		<10	80
11	<10	80	320		10	320
12	<10	20	160		20	80
13	10	<10	40		¶	¶

*HI, hemagglutination inhibition; ID, identification. †Strain A/Swine/Iowa/79 (H1N1). ‡Strain A/Swine/Texas/98 (H3N2). §Strain A/Kansas/13/2009 (H3N2). ¶Ten of the original 13 swine were slaughtered before convalescent-phase serum could be collected.

Influenza matrix RT-PCR and virus isolation on nasal swab samples were negative. HI assays demonstrated little or no antibody against the influenza (H1N1) indicator virus and low-level antibody reaction against the prototype swine influenza virus (H3N2). However, HI titers against the “county fair” influenza virus (H3N2) showed consistently elevated titers, which suggested that the animals might have been exposed to the virus 2 weeks earlier, during the time of the fair. The swine may have cleared the virus by the time the nasal swabs were collected, but without positive RT-PCR or virus isolation results, the situation remain inconclusive.

We compared the HA gene segment of A/Kansas/13/2009 (H3N2) with recent animal and human influenza (H3N2) viruses by using the neighbor-joining method, and it clustered with the HA from recent triple-reassortant SIV (H3N2) isolates (Figure A1) (*10*). A/Kansas/13/2009 (H3N2) shares >97% nucleotide identity with 2 swine viruses reported to have caused human infections, A/Ontario/RV1273/2005 and A/Ontario/1252/2007, and >90% nucleotide identity with currently circulating seasonal (H3N2) viruses, such as A/Perth/16/2009. Sequence analysis for the remaining 7 gene segments confirmed A/Kansas/13/2009 as triple-reassortant SIV (H3N2), belonging to the same lineages as the genes for the reference virus A/swine/Texas/98 (data not shown). A full genome sequence for A/Kansas/13/2009 (H3N2) has been submitted to GenBank (accession nos. GU937743–GU937750).

This case emphasizes the importance of epidemiologic and laboratory surveillance in areas where humans and swine are in close contact. When novel influenza A infection occurs in humans, joint investigations by local, state, and federal public health and animal health agencies are key to determining the source of infection and extent of transmission. The worldwide spread of pandemic (H1N1) 2009 virus emphasizes the ongoing public health threat of interspecies influenza transmission. Improved surveillance among swine may lead to early identification of novel viruses with pandemic potential and provide early opportunities to implement control measures.

## Figures and Tables

**Appendix:**
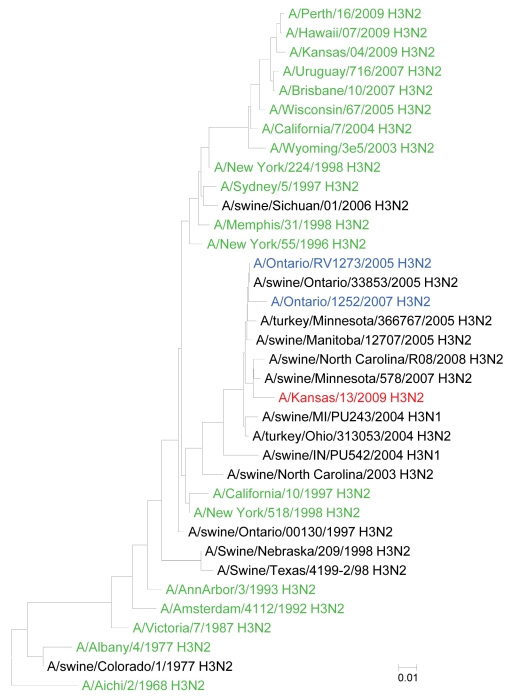
Figure. Phylogenetic tree of the hemagglutinin (HA) gene segment of influenza A (H3) viruses created by using the neighbor-joining method, nucleotide model of Kimura-2 parameters, in MEGA version 4 (*10*). In addition, a subset of H3 HA gene sequences available in the public domain was included in the analyses for comparison. Red, A/Kansas/13/2009 (H3N2); green, human (H3N2) viruses; black, animal (H3N2) viruses; blue, human cases of swine (H3N2) viruses.
